# Non-thyroidal Illness Syndrome (NTIS) is no independent predictor for mortality in ICU patients

**DOI:** 10.1186/s12871-023-02015-1

**Published:** 2023-03-31

**Authors:** Natalie Krug, Sven Bercker, Thilo Busch, Steffen Friese, Nora Jahn, Maria Theresa Voelker

**Affiliations:** 1 Department of Anesthesiology and Critical Care Medicine, University Hospital Leipzig, Liebigstr. 20, 04103 Leipzig, Germany; 2 Present Address: Clinical Department of General Anaesthesiology, Emergency and Intensive Care Medicine, LKH-University Hospital of Graz, Medical University of Graz, Graz, Austria

**Keywords:** Critical illness, Thyroid hormones, Thyroid axis, Intensive care

## Abstract

**Background:**

Low T3-(/T4-) syndrome, also known as non-thyroidal Illness Syndrome (NTIS) describes a decrease in free serum thyroid hormones without a concomitant increase in TSH, frequently observed in critically ill patients. However, whether NTIS is only a metabolic adaption to stress in critically ill or plays a crucial role as an independent risk factor for ICU mortality, remains unknown. Our study aimed to evaluate NTIS as an independent risk factor for increased ICU mortality.

**Methods:**

All patients admitted to the interdisciplinary intensive care unit (ICU) at the University Hospital of Leipzig between 2008 and 2014 were retrospectively analyzed for thyroidal function. Baseline data, information on additional thyroid function tests, disease progression, hospital and ICU length of stay (LOS) and patient outcome were retrospectively analyzed from the hospitals digital information system. For statistical evaluation, univariate analysis, matched pairs analysis and multivariate logistic regression were conducted.

**Results:**

One thousand, seven hundred ninety patients were enrolled in the study, of which 665 showed NTIS. Univariate analysis revealed a positive association of NTIS with ICU- and hospital-LOS, need for mechanical ventilation, incidence of sepsis, acute respiratory distress syndrome, acute liver failure and increased ICU mortality. Results of matched pair analysis confirmed these findings. In multivariate logistic regression, NTIS was associated with an increased ICU-LOS, increased duration of mechanical ventilation, acute kidney injury and liver failure, but showed no independent association with increased ICU-mortality.

**Conclusion:**

Duration of mechanical ventilation as well as incidence of acute kidney injury, sepsis and acute liver failure were detected as independent predictors of mortality in patients with NTIS. NTIS itself was no independent predictor of increased ICU-mortality.

**Supplementary Information:**

The online version contains supplementary material available at 10.1186/s12871-023-02015-1.

## Introduction

Non-thyroidal Illness Syndrome (NTIS) or Low T3-(/T4-) Syndrome describes a decrease in free serum thyroid hormones without a corresponding increase in thyroid stimulating hormone (TSH) in the absence of manifest thyroidal illness [1, 2]. It is a phenomenon frequently seen in critically ill patients and is associated with several severe complications such as sepsis, multiple organ failure, prolonged mechanical ventilation, extended stay on intensive care unit (ICU), and increased mortality [2–8]. The entire pathogenesis of NTIS is not yet completely understood, but it seems to be induced by pro-inflammatory cytokines, increased levels of endogenous or exogenous corticosteroids and certain drugs [3–5, 9]. Subsequently, the conversion of thyroxine (T4) to triiodothyronine (T3) in the tissue is inhibited [9], resulting in an accompanying increase of reverse triiodothyronine (rT3). Affected patients may display different types of NTIS, with NTIS Low fT3 presenting with an isolated decrease of free triiodothyronine (fT3) alone, and NTIS Low fT3 fT4 presenting with decrease in both, fT3 and free tetraiodothyronine (fT4) respectively. Initially, in critically ill, NTIS Low fT3 is the predominant entity [5, 10, 11], whereas prolonged critical illness results in NTIS Low fT3 fT4 [11]. The association between NTIS and sepsis, multiple organ failure, acute kidney injury and acute liver failure has been described previously [3–6]. However, if NTIS represents an independent risk factor for increased ICU-mortality or if it is merely a secondary effect of critical illness, is not yet well established. Therefore, in our current study we aimed to evaluate NTIS as an independent predictor for increased ICU-mortality. Furthermore, we focused on identifying potential risk factors associated with the development of NTIS in a large cohort of critically ill patients at our interdisciplinary operative ICU.

## Materials and methods

### Study design and inclusion

We conducted a retrospective observational cohort study in patients admitted to the interdisciplinary operative ICU at the University Hospital of Leipzig between December 2008 and December 2014. All patients were screened for existing data on serum levels of TSH, fT3 and fT4, which are tested routinely once per week according to our ICU protocol. Patients younger than 18 years, an ICU length of stay (LOS) of less than 24 h, any history of thyroidal or pituitary disease as well as medication affecting thyrotrophic axis (e.g. thyroidal hormone replacement, thyrostatic therapy) were excluded from the study. The study protocol was approved by the ethics committee of the Medical Faculty of the University of Leipzig (reference number: 362/18-ek).

### Data collection and parameters

Baseline parameters included age, gender, severity of disease and body mass index (BMI). For severity of disease, we registered Simplified Acute Physiology Score II (SAPS II) at the timepoint of admission to ICU. Patients were screened for the occurrence of sepsis, pneumonia, acute respiratory distress syndrome, acute liver failure and acute kidney injury (AKI) as well as the need for renal replacement therapy and mechanical ventilation. All data were retrospectively collected from the hospitals´ information system (SAP, SAP SE, Walldorf, Germany; COPRA, COPRA System GmbH, Berlin). NTIS was assumed, when fT3 and/or fT4 levels were decreased, with normal or decreased levels of TSH. Patients with known diagnosis of any thyroid illness were excluded. NTIS was classified into two subgroups as follows: the “NTIS Low fT3” group with low serum level of fT3, normal serum level of fT4 and low or normal serum level of TSH, and the “NTIS Low fT3 fT4” group with low serum level of fT3 and fT4 and low or normal serum level of TSH. Patients with any other patterns of discordant thyroid function, were grouped into the Non-NTIS cohort. The normal range of serum hormone concentrations at our laboratory were as follows: fT3: 3.1 – 6.9 pmol/l; fT4: 12.8 – 20.4 pmol/l; TSH: 0.4 – 3.77 mU/l. Quantitative serum thyroid hormone levels were analyzed by immune-assay method by electrochemiluminiscence -immunoassay (*ECLIA*) from ROCHE (Basel, Switzerland).

### Statistical evaluation

With regard to baseline data, continuous variables are reported as median with interquartile range (MD; IQR) whereas categorical variables are presented as whole numbers and percentages (%). Univariate analyses and matched pair analyses were performed according to the above-named groups. In particular, NTIS patients were matched with Non-NTIS patients according to age, gender, BMI and SAPS II. Differences between these groups were compared in a multivariate analysis. All numeric parameters were tested by Kolmogorov–Smirnov-Tests for normal distribution and are expressed as median (MD) and interquartile range (25% quantile – 75% quantile; IQR). Statistical univariate comparisons between patients with and without NTIS were performed using the χ^2^ test for nominal data. For the comparison of more than two groups of nominal data, a pair-wise post hoc with Z-test and Bonferroni-Correction was used. Kruskal–Wallis-test was applied for the comparison of quantitative data from more than two samples (NTIS Low fT3 fT4 vs. NTIS Low fT3 vs. Non-NTIS). For multiple comparison of pair-wise differences, we used Mann–Whitney U-test with Bonferroni-Correction. For the identification of parameters independently associated with NTIS, univariate logistic regression analysis was performed. To increase power of the statistical testing, we used matched pair analysis. Two groups of pairs were formed using a propensity score adjustment: NTIS Low fT3 and Non-NTIS, as well as NTIS Low fT3 fT4 and Non-NTIS. Matching was done, using age, gender, height, BMI and SAPS II-score at the timepoint of admission. Differences between groups were tested using the Mann–Whitney-U-Test or χ^2^-Test. Risk factors of univariate analysis with *p* values of < 0.1 were included in the multivariate analysis to identify significant independent risk factors in association with NTIS. A logistic regression analysis was performed to test for independent predictors of ICU-mortality and hospital-mortality. To characterize logistic regression, *p* value of chi-square (expected to be < 0.05), Nagelkerke’s R^2^, results of the Hosmer–Lemeshow goodness of fit statistics (expected to be > 0.05), sensitivity, and specificity of the statistical model are given. A *p*-value < 0.05 was considered statistically significant. Statistical analyses were executed with SPSS 24.0 (IBM Corp. Armonk, NY, USA).

## Results

### Univariate analysis

**Fig. 1:**
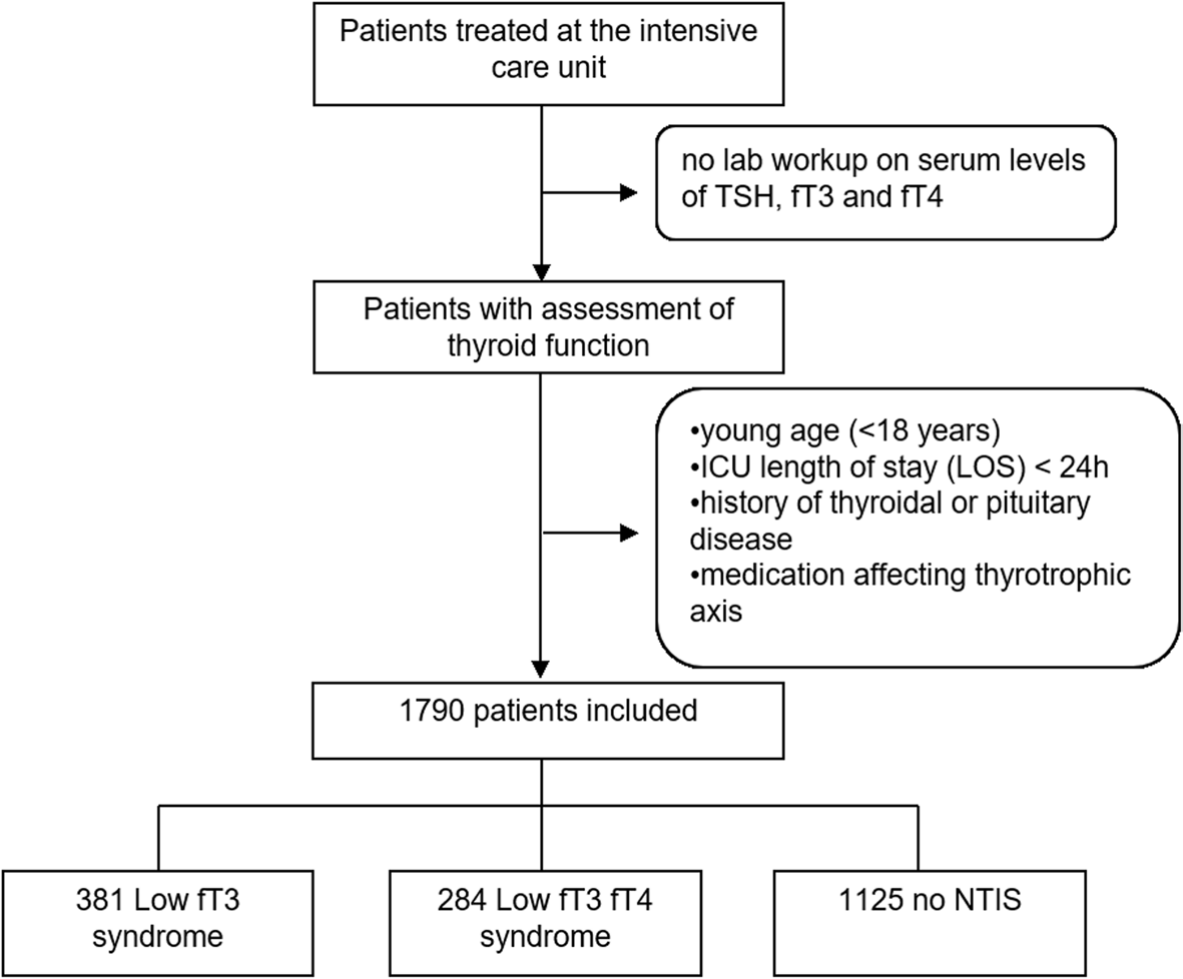
Enrolment of patients

We enrolled 1790 patients admitted to the interdisciplinary ICU of the University Hospital of Leipzig from December 2008 to December 2014, of which 665 showed NTIS (Fig. 1). 381 patients displayed a Low fT3 syndrome, and 284 patients a Low fT3 fT4 syndrome. Demographic data and characteristics are presented in Table 1. Baseline parameters were comparable between the study groups, aside from differences in age and SAPS II score. All monitored medical parameters and co-morbidities were significantly different between the study groups displaying more severely ill patients in both NTIS groups (SAPS II *p* < 0.001 vs. Non-NTIS) and an increased incidence of co-morbidities in the NTIS groups (see Table 1). Hospital mortality in patients with NTIS Low ft3 ft4 was 40.8% compared to 27.6% in NTIS Low ft3 and 16.4% in non-NTIS patients.

**Table 1:** Univariate analysis and Baseline parameters of all three groups

	Non-NTIS-group(n=1125)	NTIS Low fT3-group(n=381)	NTIS Low fT3 fT4-group(n=284)	P Kruskal Wallis/ Chi²
**baseline parameters**				
age, years; MD (IQR)	59 ( 44-72)	68 (56-77)*	61 (52-74)*§	< 0,001
male; n (%)	694 (62)	217 (57)	176 (62)	0.235
height, cm; MD (IQR)	172 (165-180)	170 (165-178)	174 (168-180)§	0.019
weight, kg; MD (IQR)	75 (65-85)	75 (65-85)	75 (66-88)	0.526
BMI, kg/m²; MD (IQR)	25 (23-28)	25,4 (23-28)	25,2 (23-29)	0.951
SAPS II at admission; MD (IQR)	33 (22-48)	41 (32-56)*	44 (32-57)*	< 0,001
**thyroid hormones**				
TSH, mU/l; MD (IQR)	2,24 (0,54-4,87)	0,49 (0,23-1,33) *	0,63 (0,23-1,88) *	< 0,001
fT3, pmol/l; MD (IQR)	3,86 (3,20-4,73)	2,47 (2,12-2,80) *	2,10 (1,62-2,54) *§	< 0,001
fT4, pmol/l; MD (IQR)	16,3 (13,9-19,1)	15,7 (14,1-17,3)	10,4 (9,0-11,7) *§	< 0,001
**diagnosis, procedures**				
sepsis; n (%)	106 (9)	62 (16) *	99 (35) *§	< 0,001
pneumonia; n (%)	140 (12)	76 (20)	99 (35) *§	< 0,001
ARDS; n (%)	35 (3)	21 (6) *	37 (13) *§	< 0,001
mech. ventilation; n (%)	653 (58)	281 (74) *	252 (89) *§	< 0,001
mech. ventilation, hrs; MD (IQR)	38 (5-211)	147 (18-368) *	198 (58-485) *§	< 0,001
AKI; n (%)	99 (9)	64 (17)*	105 (37) *§	
liver disease; n (%)	237 (21)	89 (23)	93 (33) *§	< 0,001
liver failure; n (%)	41 (4)	30 (8) *	47 (17) *§	< 0,001
intracranial hemorrhage/ lesion; n (%)	360 (32)	91 (24) *	72 (25)	0.003
diabetes mellitus; n (%)	226 (20)	111 (29)*	72 (25)	0.001
**length of stay, mortality**				
ICU-LOS, d; MD (IQR)	8 (3-18)	15 (6-28) *	23 (10-40) *§	<0,001
hospital-LOS, d; MD (IQR)	21 (11-36)	28 (17-45) *	32 (19-59) *	<0,001
ICU-mortality; n (%)	155 (14)	75 (20)*	105 (37) *§	<0,001
hospital-mortality; n (%)	185 (16)	105 (28)*	116 (41) *§	<0,001

*NTIS* Non-thyroidal Illness Syndrome, *BMI* Body mass index, *SAPS II* Simplified acute physiology score, *MD* Median, *IQR* Interquartile range, *n* Absolute frequency, *TSH* Thyroid stimulating hormone, *fT3* Triiodothyronine, *fT4* Thyroxine, *ARDS* Acute respiratory distress syndrome, *hrs* Hours, *AKI* Acute kidney failure, *ICU* Intensive care unit, *LOS* Length of stay, *d* Days

^*^ *p* < 0.05 vs. Non-NTIS; §: *p* < 0.05 vs. NTIS Low fT3

### Matched pair analysis

Three hundred eighty one Non-NTIS patients were matched to 381 NTIS Low fT3 patients, and 284 Non-NTIS patients were matched to the same number of patients with NTIS Low fT3 fT4 (Table 2). Matched pair analysis revealed a significantly higher incidence of sepsis, ARDS, duration of mechanical ventilation, acute liver failure, ICU- and hospital-LOS for NTIS Low fT3 and NTIS Low fT3 fT4 patients compared to Non-NTIS patients (Table 2). NTIS Low fT3 fT4 patients showed a higher incidence of mechanical ventilation, acute kidney injury, and ICU- and hospital mortality compared to patients without NTIS (Table 2). The difference in mortality between NTIS Low fT3 and Non-NTIS patients observed in univariate analysis, could not be confirmed in matched pair analysis (Non-NTIS ICU mortality of 22.3% vs. 27.6% in NTIS Low fT3, *p* = 0.09). Incidence of intracranial hemorrhage/lesions and diabetes were comparable between the study groups.

**Table 2:** Matched pair analysis of Non-NTIS vs. NTIS Low fT3 and Non-NTIS vs. NTIS Low fT3 fT4

	Non-NTIS-group(n=381)	NTIS Low fT3-group(n=381)	Non-NTIS-group(n=284)	NTIS Low fT3 fT4-group(n=284)
**Baseline parameters**				
age, years; MD (IQR)	67 (56-75)	68 (56-77)	63 (52-75)	61 (52-74)
male; n (%)	214 (56)	217 (57)	176 (62)	176 (62)
height, cm; MD (IQR)	170 (165-176)	170 (165-178)	172 (165-178)	174 (168-180)
weight, kg; MD (IQR)	75 (65-85)	75 (65-85)	75 (65-85)	75 (66-88)
BMI; MD (IQR)	25 (23-28)	25 (23-28)	26 (23-30)	25 (23-29)
SAPS II; MD (IQR)	43 (30-57)	41 (32-56)	43 (29-56)	44 (32-57)
**diagnosis, procedures**				
sepsis; n (%)	39 (10)	62 (16) *	34 (12)	99 (36) *
mechanical ventilation; n (%)	244 (64)	281 (74) *	52 (18)	99 (35) *
mechanical ventilation, h; MD (IQR)	53 (7-182)	147 (18-368) *	11 (4)	37 (13) *
pneumonia; n (%)	62 (16)	76 (20)	189 (67)	252 (89) *
ARDS; n (%)	9 (2)	21 (6) *	56 (7-202)	198 (58-485) *
AKI; n (%)	48 (13)	64 (17)	38 (13)	105 (37) *
liver disease; n (%)	98 (26)	89 (23)	69 (24)	93 (33) *
liver failure; n (%)	14 (4)	30 (8) *	14 (5)	47 (17) *
intracranial hemorrhage and lesion; n (%)	123 (32)	91 (24) *	89 (31)	72 (25)
diabetes mellitus; n (%)	95 (25)	111 (29)	74 (26)	72 (25)
**length of stay, mortality**				
ICU-LOS, d; MD (IQR)	10 (5-23)	15 (6-28) *	9 (4-22)	23 (10-40) *
hospital-LOS, d; MD (IQR)	24 (15-40)	28 (17-45) *	22 (13-41)	32 (19-59) *
ICU-mortality; n (%)	69 (18)	75 (20)	55 (19)	105 (37) *
hospital-mortality; n (%)	85 (22)	105 (28)	63 (22)	116 (41) *

*NTIS* Non-thyroidal Illness Syndrome, *MD* Median, *IQR* Interquartile range, *n* Absolute frequency, *BMI* Body mass index, *SAPS* Simplified Acute Physiology Score, *ARDS* Acute Respiratory Distress Syndrome, *AKI* Acute kidney injury, *ICU* Intensive care unit, *LOS* Length of stay

^*^ *p* < 0.05 vs. non-NTIS

### Multivariate logistic regression

**Fig. 2:**
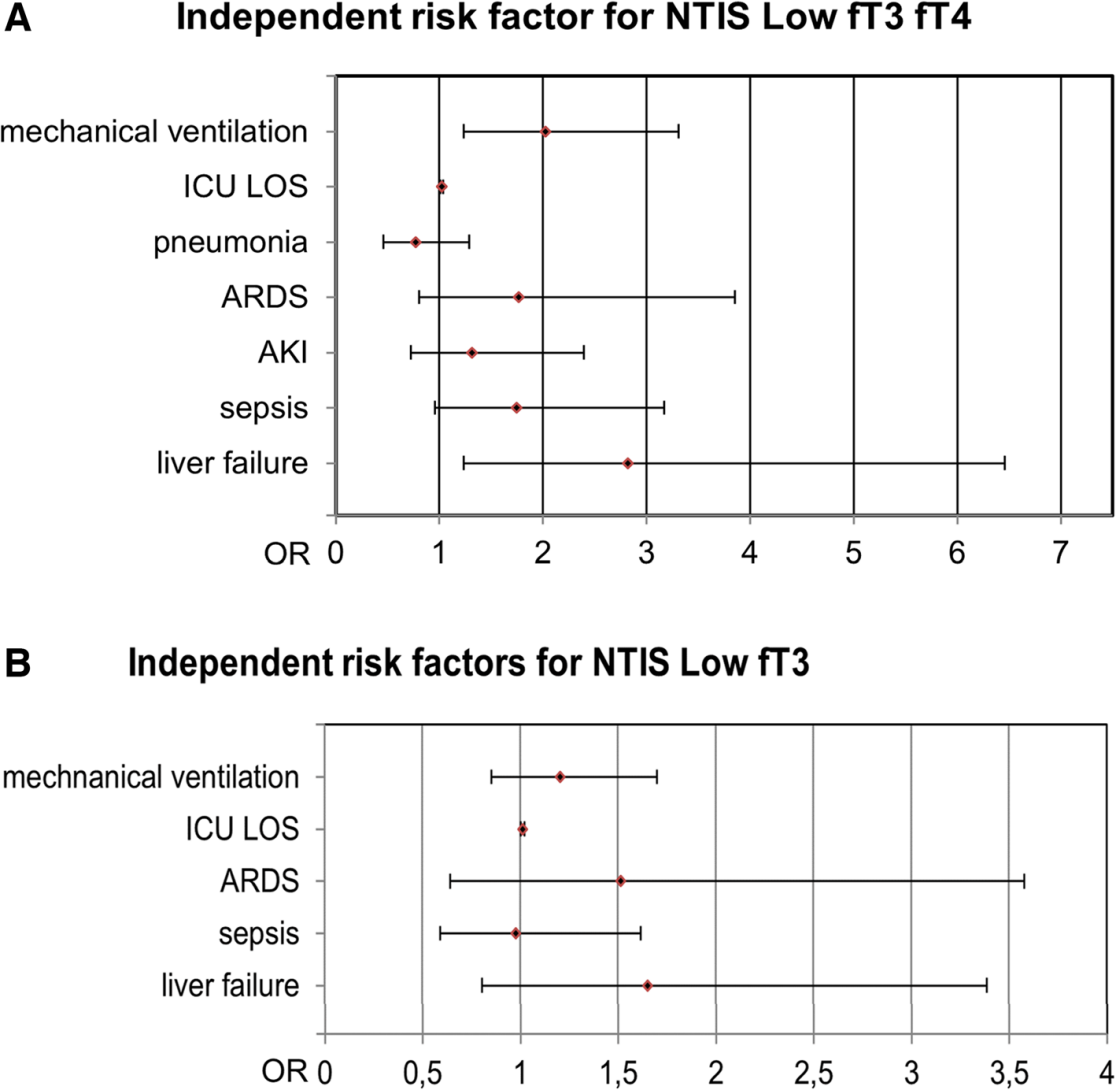
Independent risk factor for NTIS Low fT3 and NTIS Low fT3 fT4. Multivariate logistic regression of risk factors for NTIS Low fT3 fT4 (**A**) and NTIS Low fT3 (**B**); forest plot; full data may be found in the supplements section (Suppl. Tables 1 and 2); ICU: intensive care unit; LOS: length of stay; ARDS: Acute Respiratory Distress Syndrome

Multivariate analysis was performed for age, SAPS II, mechanical ventilation, pneumonia, ARDS, AKI, sepsis, acute liver failure and NTIS itself. Multivariate analysis revealed ICU-LOS (OR 1.013; 95% CI 1.005–1.022; *p* = 0.002) as an independent predictor for NTIS Low fT3 (Fig. 2 B; Suppl. Table 1). Mechanical ventilation (OR 2.022; 95% CI 1.235–3.309; *p* = 0.005), ICU-LOS (OR 1.023; 95% CI 1.012–1.034; *p* < 0.001) and acute liver failure (OR 2.823; 95% CI 1.235–6.456; *p* = 0.014) were identified as independent predictors for NTIS Low fT3 fT4 (Fig. 2 A, Table 2).

**Fig. 3:**
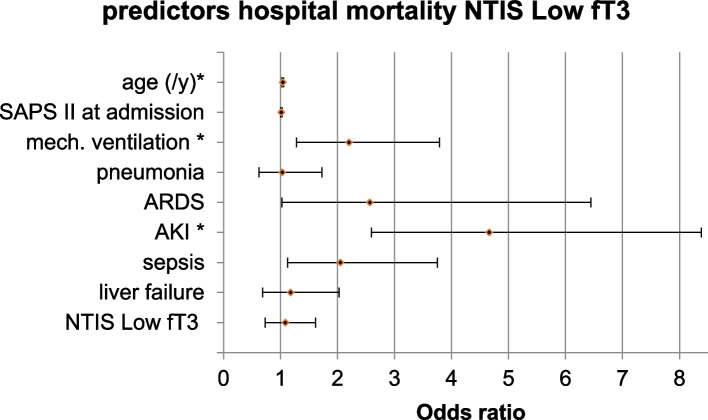
Hospital mortality NTIS Low fT3. Multivariate logistic regression of risk factors for hospital mortality of patients with NTIS Low fT3; Forest blot; binary logistic regression including *n* = 381 patients with NTIS Low fT3. SAPS: Simplified Acute Physiology Score; mech.: mechanical; ARDS: Acute Respiratory Distress Syndrome; AKI: acute kidney injury; ICD-10: International Classification of Diseases

**Fig. 4:**
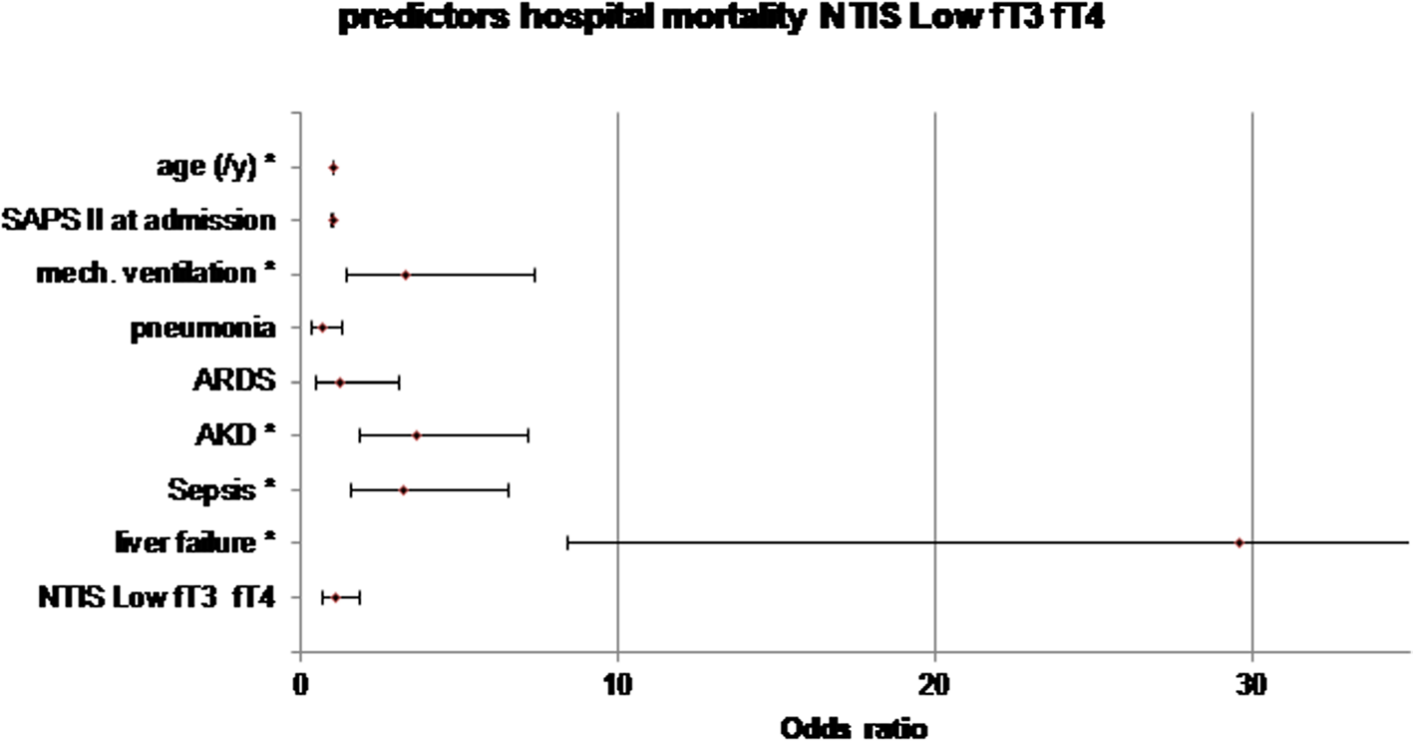
Hospital mortality NTIS Low fT3 fT4. Multivariate logistic regression of risk factors for hospital mortality of patients with NTIS Low fT3; Forest blot; odds ratio for liver failure 29.6 (lower value 8.3; upper value 104.0) binary logistic regression including *n* = 284 patients with NTIS Low fT3. SAPS: Simplified Acute Physiology Score; mech.: mechanical; ARDS: Acute Respiratory Distress Syndrome; AKI: acute kidney injury; ICD-10: International Classification of Diseases

Evaluation of in-hospital mortality in patients with NTIS Low fT3 revealed all analyzed parameters as independent predictors of increased mortality, except pneumonia and NTIS itself with a sensitivity of 44.7% and specificity of 94.6% (Table 3, Figs. 3 and 4). For patients with NTIS Low fT3 fT4, independent predictors for hospital mortality did not differ from NTIS Low fT3, except for ARDS, with no independent risk increase (Table 3). Sensitivity was higher for NTIS Low fT3 fT4 compared to NTIS Low fT3 (sensitivity of 63.1%, specificity of 93.8%, Table 3). In logistic regression, the development of NTIS was no significant predictor for hospital mortality in any of the investigated groups (Table 3).

**Table 3:** Hospital mortality NTIS Low fT3 and NTIS Low fT3 fT4

	NTIS Low fT3				NTIS Low fT3 fT4			
parameters	regression coefficient	Odds Ratio	95% confidence levelfür Odds Ratio		regression coefficient	Odds Ratio	95% confidence levelfür Odds Ratio	
			lower value	upper value			lower value	upper value
age	0.039	1.040	1.022	1.057	0.049	1.050	1.030	1.071
SAPS II	0.014	1.014	1.002	1.026	0.013	1.013	0.999	1.027
mech. ventilation	0.789	2.201	1.278	3,790	1.198	3.312	1.480	7.411
pneumonia	0.032	1.032	0.618	1.725	-0.360	0.698	0.370	1.316
ARDS	0.943	2.567	1.022	6.446	0.215	1.239	0.490	3.133
AKI	1.539	4.661	2.592	8,380	1.305	3.687	1.890	7.192
sepsis	0.718	2.051	1.122	3.749	1.178	3.247	1.599	6.592
liver failure	1.328	3.772	1.408	10.104	3.387	29.581	8.425	103.860
NTIS	0.080	1.084	0.728	1.614	0.115	1.122	0.670	1.878
constant	-5.587				-6.545			

Multivariate logistic regression of risk factors for hospital mortality of patients with NTIS Low fT3; binary logistic regression including *n* = 381 patients with NTIS Low fT3 and *n* = 284 patients with NTIS Low fT3 fT4 after Propensity-Score adjustment (in terms of age, gender, height, body mass index and SAPS at admission ICU)

*SAPS* Simplified Acute Physiology Score, *mech* Mechanical, *ARDS* Acute Respiratory Distress Syndrome, *AKI* Acute kidney injury, *ICD-10* International Classification of Diseases; Hosmer–Lemeshow-Test

*p* = 0.740; Nagelkerke`s R^2^ 0.362/ 0.555; Modell Chi^2^ 213.14/ 327.98; *p* < 0.001; sensitivity: 44.7% (85/190)/ 63.1% (113/179) correctly predicted deceased; specificity: 94.9% (543/572)/ 93.8% (365/389) correctly predicted survivors

## Discussion

In critically ill patients, serum thyroid hormone levels are often found to be deranged in a characteristic way. This phenomenon, called NTIS (non-thyroidal Illness Syndrome) or low fT3-(/fT4-) syndrome, strongly correlates with the severity of critical disease [6]. However, underlying pathophysiological mechanisms and causality of the association are still poorly understood.

In our current retrospective study, we aimed to enlighten the possible associations between patients’ characteristics, severity of critical disease and NTIS in a large cohort of critically ill patients. Univariate analysis showed significant differences between patients with and without NTIS in numerous analyzed parameters, including age, severity of critical disease and ICU-LOS. No differences were found for gender and BMI. Our current findings are in accordance with data from a variety of former studies [3–6], however profound insight in causality and relevance of NTIS for patient morbidity and mortality is still lacking [6, 12–15]. As NTIS is primarily associated with critical illness, higher SAPS II scores and an increased number of organ failures (ARDS, acute kidney injury, acute liver failure) [6] may be expected in patients with NTIS.

Our matched pair analysis showed a significant increase in a multitude of co-morbidities as well as an inferior outcome in NTIS Low fT3 fT4 patients compared to Non-NTIS patients. These findings were less evident, when patients with isolated Low fT3 were compared to Non-NTIS patients (Table 2). Regarding the pathogenesis of NTIS, prior investigations could identify different serological inflammatory markers such as TNF-α, IFN-γ, and interleukin-6 to decrease the activity of 5’-deiodase (5’-DI) of the liver, thyroid gland and peripheral tissue [10, 16–20]. Those markers are known to rise quickly in critically ill patients, leading to a subsequent decrease in conversion and following serum concentration of fT4 to fT3 [4, 10]. The elevation of pro-inflammatory markers additionally mediates an increase of 5’DI in the pituitary gland [21]. Thereby, pituitary tissue fT3 levels are elevated [22] and the usually observed central feedback to low serum hormones with a subsequent TSH increase, is lost. This results in the NTIS-typical serological hormone pattern with normal serum TSH concentrations in combination with reduced free thyroidal hormones. Another explanation for the changes seen in NTIS might be the derangement of glucocorticoid levels (endogenous or exogenous) in critically ill patients as glucocorticoids are affecting stress response generally and the pituitary responsiveness to TRH specifically [23–25]. Due to these well-established pathophysiological changes and the observation, that NTIS Low fT3 is frequently observed during the initial phase of critical illness [3], NTIS Low fT3 is generally assumed to be a metabolic adaptive phenomenon of the thyroid function readjusting to critical illness as oppose to an outcome-relevant self-sufficient pathomechanism. With this, NTISwould not require specific medical treatment or therapy [7]. Interestingly, in our current study, NTIS Low fT3 showed only little association with co-morbidities typically seen in the ICU setting and had no significant influence on ICU-mortality in comparison to patients without NTIS. However, as the critical illness progresses, a consecutive decrease in fT4 is additionally detected, leading to a profound thyreoid hypofunction due to impaired central feedback mechanism. In our current study, these patients showed a significantly increased mortality in the matched pair analysis. Hence, one would expect the progressing NTIS to be an independent risk factor for mortality and morbidity in ICU patients, as adaptive mechanisms fail. However, in our logistic regression analysis, NTIS of either entity did not turn out to be an independent predictor of hospital mortality in our patient cohort. This underlines the preexisting hypothesis of NTIS being merely an epiphenomenon in critically ill patients, induced by inflammatory processes rather than a co-morbidity of its own that needs to be treated [26].

Although our current study shows interesting results, several limitations should be discussed. Firstly, by implication of the retrospective setup, we had no influence on the timing of laboratory workup and thyroid function testing. Secondly, critically ill patients are a generally inhomogeneous group of patients ranging from severe trauma to infections or intoxications. This data incorporates all groups of patients despite the fact, that the cause of NTIS is likely multifactorial and may also vary in different groups of patients. However, due to the very large number of patients, this might also be a strength as it may still reveal common factors. Thirdly, and probably most importantly, our study can only show associations, but no causalities. The question of causalities, hence the question, whether NTIS is either the sequel or rather one of the reasons for critical illness, may only be answered from this point on by large observational, multicenter clinical studies, correcting for known risk factors. A direct extrapolation of our results to clinical recommendations without prior evaluation in prospective studies cannot be recommended. However, such studies do not yet exist and, to our knowledge, so far, our study is the largest retrospective database analysis addressing this relevant topic.

## Conclusion

This study is the largest database study on critically ill patients with NTIS. It further enlightens the association between NTIS and a multitude of critical co-morbidities, frequently observed in ICU patients. Our central finding is that NTIS Low fT3 and NTIS Low fT3 fT4 were no independent predictors for increased mortality. This confirms the current assumption that NTIS is merely one part of the adaptation to severe illness and has—according to our current knowledge—no pathological entity of its own.

## Supplementary Information

**Additional file 1:Suppl. Table 1.** Multivariate regression, independent risk factor for NTIS Low fT3. **Suppl. Table 2.** Multivariate regression, risk factors for NTIS Low fT3 fT4.

## Data Availability

The datasets generated during and/or analysed during the current study are available from the corresponding author on reasonable request.

## Abbreviations

AKI: Acute kidney failure
ARDS: Acute respiratory distress syndrome
fT3: Triiodothyronine
fT4: Thyroxine
hrs: Hours
ICU: Intensive care unit
IQR: Interquartile range
LOS: Length of stay
MD: Median
NTIS: Non-thyroidal Illness Syndrome
SAPS: Simplified acute physiology score
TSH: Thyroid stimulating hormone
BMI: Body mass index
