# Supplementary material for: Non-thyroidal Illness Syndrome (NTIS) is no independent predictor for mortality in ICU patients
Source: BMC Anesthesiol. 2023 Mar 31;23:103. doi: 10.1186/s12871-023-02015-1 (PMC10064728; doi:10.1186/s12871-023-02015-1)
Supplement: Supplementary file 1 — Additional file 1:Suppl. Table 1. Multivariate regression, independent risk factor for NTIS Low fT3. Suppl. Table 2. Multivariate regression, risk factors for NTIS Low fT3 fT4. [file 12871_2023_2015_MOESM1_ESM.docx]

**Supplement**

Suppl. Table 1: Multivariate regression, independent risk factor for NTIS Low fT3.

ICU: intensive care unit; LOS: length of stay; ARDS: Acute Respiratory Distress Syndrome

Suppl. Table 2: Multivariate regression, risk factors for NTIS Low fT3 fT4.

ICU: intensive care unit; LOS: length of stay; ARDS: Acute Respiratory Distress Syndrome; AKI: acute kidney injury
